# Acknowledgment

**Published:** 1891-10

**Authors:** 


### Acknowledgment.

On other pages of this number of the REGISTER will be found a record of the proceedings of the National Association of Boards of Dental Examiners, and also of the Association of College Faculties; for the preparation of these reports THE REGISTER is indebted to Mr. Hise, of *The Dental Cosmos*, he having sent advanced proof sheets, for which he has our hearty thanks.

